# The effect of footwear on multi-segment foot kinematics during running

**DOI:** 10.1186/1757-1146-5-S1-O2

**Published:** 2012-04-10

**Authors:** Robin L Bauer, Mukta N Joshi, Trevor R Klinkner, Stephen C Cobb

**Affiliations:** 1 Department of Kinesiology, University of Wisconsin-Milwaukee, Milwaukee, WI 53201, USA

## Background

Footwear is intended to prevent lower extremity injuries caused by excessive foot-ground impacts and faulty mechanics. However, no clear relationship between shoe habits and injury risk has been established [1]. Many studies have examined barefoot versus shod running kinematics, but the results have been equivocal [2,3]. A factor in the inconsistent results could be the relationship between foot structure and function. For example, Cobb et al. demonstrated significant walking gait kinematic differences between participants with typical and low arch foot structures using a multi-segment foot model [4]. The purpose of this study was to investigate effects of footwear on multi-segment foot kinematics during running in participants with low arch structure.

## Materials and methods

Five healthy participants (26.8 ± 9.01 yrs; 171.5 ± 9.85 cm; 71.61 ± 15.46 kg) with low arch structure completed 10 running trials at 4.0 (±10%) m/s in flat sandal and barefoot conditions. Marker clusters placed on the skin or custom-built wands identified six functional articulations: rearfoot complex (RC), calcaneonavicular complex (CNC), calcaneocuboid joint (CC), medial forefoot (MFF), lateral forefoot (LFF), and 1st metatarsophalangeal complex (MTP). Repeated measures MANOVAs (α ≤ 0.05) were used to analyze within-subject sagittal, frontal and transverse plane range of motion (ROM) and initial contact position differences between footwear conditions for the RC, CNC, and CC articulations. Dependent t-tests (α ≤ 0.05) were performed to assess MTP, MFF and LFF articulation sagittal plane ROM differences between the footwear conditions.

## Results

ROM between conditions are shown in Table 1 and initial contact positions are shown in Table 2.

**Table 1:** ROM mean ± SD results for Sagittal, Frontal and Transverse planes of motion

	Barefoot			Flat		
	
	Sagittal Plane	Frontal Plane	Transverse Plane	Sagittal Plane	Frontal Plane	Transverse Plane
**RC**	22.16 ± 3.18	8.33 ± 1.72	16.99 ± 1.92	20.44 ± 5.30	8.10 ± 2.92	13.98 ± 2.95
	
**CNC**	10.85 ± 2.57	8.11 ± 2.67	6.35 ± 1.72	9.39 ± 4.02	6.14 ± 4.33	7.44 ± 2.35
	
**CC**	18.66 ± 1.86	9.69 ± 2.44	8.88 ± 2.92	14.48± 5.35	7.02 ± 2.75	7.45 ± 1.45
	
**MFF**	20.90 ± 4.68			20.14 ± 3.60		
**MTP**	41.35 ± 5.39*			33.33 ± 1.76*		
**LFF**	7.68 ± 3.19			9.34 ± 2.52		

*indicates a significant difference (p < 0.05) between footwear conditions.

**Table 2:** Significant initial contact positions (p < 0.05) mean ± SD results between footwear conditions.

	Barefoot			Flat		
	
	Sagittal Plane	Frontal Plane	Transverse Plane	Sagittal Plane	Frontal Plane	Transverse Plane
**CNC**	2.81 ± 4.17	.38 ± 2.52	-1.53 ± 3.03*	1.51 ± 2.67	.07 ± 5.22	-4.34 ± 3.38*
	
**CC**	5.46 ± 7.60	-2.08 ± 2.84	-3.37 ± 3.23*	3.08 ± 4.03	2.60 ± 4.26	2.50 ± 1.97*
	
**MTP**	-14.12 ± 10.38*			-2.54 ± 7.63*		

*indicates a significant difference (p < 0.05) between footwear conditions.

## Conclusions

Runners alter their gait from shod to barefoot running. The ROM differences suggest runners adapt by increasing motion during stance phase. Initial contact positions demonstrate differences in strike pattern. Higher sagittal plane values for barefoot trials may indicate more midfoot/forefoot landing. These data may enhance the understanding of shoe-wear and running-related injuries.

## References

[B1] Wen D Risk factors for overuse injuries in runners Curr Sports Med Rep 2007 6 307 313 10.1007/s11932-007-0067-y 17883966

[B2] De Wit B Clercq D Aerts P Biomechanical analysis of the stance phase during barefoot and shod running J Biomech 2000 33 269 278 10.1016/S0021-9290(99)00192-X 10673110

[B3] Lieberman D Venkadesan M Werbel W Daoud A Andrea S Davis I Mang’Eni R Pitsiladis Y Foot strike patterns and collision forces in habitually barefoot versus shod runners Nature 2010 463 531 535 10.1038/nature08723 20111000

[B4] Cobb S Tis L Johnson J Wang Y Geil M McCarty F The effect of low-mobile foot posture on multi-segment medial foot model gait kinematics Gait Posture 2009 30 334 339 10.1016/j.gaitpost.2009.06.005 19615908

